# Modern techniques of teaching and learning in medical education: a descriptive literature review

**DOI:** 10.15694/mep.2021.000018.1

**Published:** 2021-01-21

**Authors:** Krishna Teja Challa, Abida Sayed, Yogesh Acharya

**Affiliations:** 1 Avalon University School of Medicine; 2 Western Vascular Institute. Galway University Hospital

**Keywords:** Education, Medical, Teaching, Learning, Problem-Based Learning, Evidence-Based Medicine.

## Abstract

This article was migrated. The article was marked as recommended.

Objectives:

Education is a dynamic process that has to be refined periodically. Lack of innovative teaching techniques in academics makes medical curricula inadequate in making a significant stride towards the future. The objective of this review is to describe and assess alternative methods of teaching and learning which can be supplementive or alternative to traditional lectures for promoting active student participation and smooth flow of information.

Methods:

A review of literature is performed with PubMed and EBSCO using the keywords: “learning” OR “didactic learning” OR “alternative learning” OR “modern learning techniques” AND “medical education”. Databases were searched and 500 studies were identified out of which 200 were selected for further screening based on inclusion criteria and exclusion criteria. Articles were surveyed based on their relevance and significance to our study objectives with both qualitative and quantitative studies were examined.

Results:

Case-based learning, evidence-based medicine, problem-based learning, simulation-based learning, e-learning, peer-assisted learning, observational learning, flipped classroom and team based learning are some of the modern learning methodologies. The various learning methods discussed attend to individual learning differences allowing students to broaden their thinking and professional knowledge by improving logical and critical thinking, clinical reasoning, and time management. Early introduction of integrative approaches develop student competency and leadership equipping students for a smooth transit into the clinical practice.

Conclusion:

This study highlights the importance and challenges of modern learning systems. With technological advancement and wider implications of medical information, students require innovative skills through inter-professional learning. It is necessary to introduce and implement flexible medical curricula that accommodates distinct modern teaching to effectively balance and bridge the gap between traditional teaching methodologies and modern educational requirements.

## Introduction

Didactic lectures (DL) have been the gold standard and the most common method of traditional teaching and learning practice. DL depends on the instructor, who teaches large amounts of information with minimal student engagement, and are typically conducted in an instructor - centered classroom, centralizing the knowledge, content, and involvement of students (Cortazzi *et al.,* 2001). Despite traditional preferences for simplicity of lecture presentations, appropriateness for crowded classes, and the ability to present massive amounts of theoretical content, students are exposed to large amounts of information making it difficult to retain, remember, and interpret (Papanna *et al.,* 2013; Jiraporncharoen *et al.,* 2015). But learning is an active process, in which the students and teachers have to work mutually to make this knowledge-sharing process enjoyable and easier for comprehension. For effective learning, teaching should facilitate development of analytical approaches to a problem and address critical areas. Students should be able to use knowledge and skills obtained in the class to satisfy their professional goals while being equipped with different learning styles and having the opportunity for feedback and discussions on their learning process thereby enhancing students’ learning effects ( Sudarso, Rahayu and Suhoyo, 2016). Thus, it becomes essential to utilize an approach to teaching and learning that best meets the specific needs of the students ( Saville *et al.,* 2006). For this reason, modern education systems should encompass multiple alternative teaching and learning strategies which are well validated and applicable to a typical classroom setting in medical schools.

The objective of this paper is to discuss and analyze different teaching-learning alternatives in modern medical education.

## Methods

A review of literature from ‘PubMed’ and ‘EBSCO’ was performed using the keywords: “learning” OR “didactic learning” OR “alternative learning” OR “modern learning techniques” and “medical education”. Databases were searched and 500 studies were identified out of which 200 were selected for further screening based on inclusion criteria and exclusion criteria. Inclusion criteria was set to include studies that were peer-reviewed, published between year 2000-2020, articles reported in English, abstracts that contain at least one of the key search terms. Exclusion criteria was set as literature in non-english languages, studies published before 1990, little or no focus on educational methodologies, studies that did not include at least one of the key search terms.

## Results

We identified nine different types of modern learning techniques through our literature search. These modern techniques of teaching and learning are well validated and commonly being employed in different parts of the world to fulfil specific curricular objectives. We carefully reviewed them to analyze their perceived and proven effectiveness, and challenges in introduction and implementation. Similarly, we also provided recommendations to overcome these limitations/challenges.

Modern Techniques of teaching and learning:

### 1. Case based learning (CBL)

CBL is a teaching-learning practice where clinical cases are employed to aid in traditional lectures. CBL promotes active learning, and is being used recently to compensate for the lack of motivation in didactic lectures. Students are given the opportunity to explore real cases in which patient history, signs, symptoms along with clinical and laboratory findings are provided ( Singhal, 2017). Through teamwork and peer interaction, students assess the case while planning for investigations and appropriate management. The motto is to equip students with the necessary aptitude required for critical analysis.

CBL links theory to practice promoting inquiry-based learning techniques. Students are put into groups and presented with clinical cases to simulate real life scenarios. These groups of students then work as a team to discuss and analyze the case to uncover differential diagnoses, management strategies, and future plans. CBL covers large amounts of topics with clear learning objectives and enhances clinical knowledge, improved teamwork, clinical skills, and practice-based behaviour ( Zinski *et al.,* 2017; McLean *et al.*, 2016).

#### 1.1 Limitations:

Although CBL can be effective in promoting group discussions, proper measures have to be taken in organizing the groups. Students and teachers require additional time to prepare for each session. This directly interferes with the student’s timetable and exam preparation. Furthermore, some instructors’ attitude, personality and disposition lead to dominance of the process with little time for independent student exploration of the case interfering with experiential learning.

#### 1.2 Recommendations:

Instructors need to undergo proper training to effectively utilize and appreciate CBL. This learning technique is more effective when conducted through small group sessions, with the involvement of engaged learners and cases closely related to clinical scenarios ( Thistlethwaite *et al.*, 2012). Furthermore, MCQ format assessments can be used to screen and monitor student understanding and thought process to ensure that students effectively utilize this learning strategy.

### 2. Evidence based medicine (EBM)

EBM provides students with the necessary tools to learn, comprehend, and appraise medical literature. EBM follows five steps: a) translation of undetermined information to an answerable question, b) retrieval of the best evidence available, c) critical comprehension of evidence for internal validity, d) application of results in practice, and e) performance evaluation. It advocates long-lasting learning and disciplined thinking by allowing meticulous and sensible application of current medical evidence in decisions regarding patient care ( Gagliardi, Stinnett and Schardt, 2012).

Although EBM has been compared to the difference between experimental and personal equipoise, the early introduction of EBM in medical schools has been effective in changing the thought process of medical graduates. Students are better equipped with analytical ability and decision-making capacity with positive impact on building competency. Implementing EBM into the conventional medical curriculum improves students’ research knowledge, personal application, outlook, and future use of the learned methods ( Ma *et al.*, 2014). In comparison with the integration of EBM along with traditional medical curricula, the modern techniques of learning medicine shows a holistic approach promoting innovation and spontaneity. It also increases the ability for logical and critical thinking, better suited for the understanding of disease background and subsequent management ( Sánchez-Mendiola *et al.*, 2012).

#### 2.1 Limitations:

Despite its indispensable role in modern education, incorporation of EBM has to be properly guided into the medical curriculum to achieve its objectives. It is to be seen precisely through the filter of personal training and experience as reflective learning is the best predictor of an optimal outcome. Insufficiency of knowledge and experience within the research field are some of the challenges. Students need to become acquainted with computers and have to undergo proper training on how to execute effective research using online databases. Similarly, statistical understanding can be challenging at times limiting its implementation.

#### 2.2 Recommendations:

EBM should be amply covered and supported by both undergraduate and postgraduate education to remove obstacles thereby preparing students with the necessary skills allowing for the effective utilization of this learning strategy. It should be introduced early in the course curriculum to develop analytical reasoning ability through the appraisal of the medical literature. Small assessments can be administered prior and after EBM training to better understand the principles of EMB. Monitoring EBP practice in clinical programs is a priority to ensure that students utilize EBM appropriately and reflect in their clinical practice (Ubbink, Guyatt and Vermeulen, 2012).

### 3. Problem based learning (PBL)

PBL is a modern learning system which combines complementary educational principles in the form of a clinical problem. It is particularly aimed at improving the quality of educational outcomes through collaborative, integrated, self-directed and comprehensive learning. An important and basic tenet of PBL is “problem first” learning, in which students attempt to solve a problem without receiving formal lectures on the subject matter. Generally, delivery of PBL is done through small group tutorials in which instructions are relayed by the teacher serving as a facilitator. These tutorials typically consist of various sessions, each dedicated to a problem in which a self-study period is allocated for searching and gathering information. This creates opportunities for students to pursue and lay a firm foundation of self-directed learning. Medical students are required to co-construct their own meaning and understanding of reflective knowledge through social interaction rather than having pre-synthesized knowledge passively conveyed to them.

PBL is acknowledged as a powerful strategy supporting higher-order cognitive processes among the members of the group (Donner and Bickley, 1993). Knowledge application and diagnostic reasoning skills are acquired through given cases to address a variety of clinical problems ( Tshitenge, Ndhlovu and Ogundipe, 2017). Students develop better clinical reasoning skills, use time efficiently, and retain clinical knowledge. Such skills and attributes are especially important for subsequent practice as the need for continuing medical education becomes widely accepted, and necessary to cope with the explosion of medical information and technology.

#### 3.1 Limitations:

Periodic evaluation of students’ performance during PBL sessions can be challenging, necessitating the definitive methods of evaluation. Students should also have simultaneous access to the equivalent resources to be able to have the same advantages as their fellow colleagues. However, information overload can become an obstacle in effectively completing the case objectives. Furthermore, the adoption of PBL will require students to make a conscious effort to assume responsibility, in order to attain better performance in the examinations.

#### 3.2 Recommendations:

The success of the PBL depends on the cooperation between the student and teacher. Students are required to participate in class already having prepared the particular subject, allowing them to clear any misconceptions and gaps in their knowledge. Students should also be encouraged to conduct peer assessments in an effort to enhance peer learning. Furthermore, successful PBL employment can occur with an inter-professional approach centred on teamwork, collaboration, and assured professional identity, therewith shaping professional growth ( Redshaw and Frampton, 2014).

### 4. Simulation based learning (SBL)

Simulation represents a man-made illustration of a true world to attain instructional motives through experiential learning. The main principle behind simulation learning is to utilize simulation aids to mimic real clinical scenarios. Although medical simulation is quite new, simulation has been used for a long time in other high risk professions such as aviation. Medical simulation permits the retrieval of clinical skills through intended practice rather than an apprentice-style of learning. It can assist as a substitute to real patients and clinical scenarios. Barriers that surround limited clinical settings have encouraged the use of SBL into preclinical teaching. One of the most important advantages is the absolute freedom for trainees in making and repeating mistakes without harming the patient ( Mulugeta *et al.*, 2018).

Virtual reality can also be implicated into SBL to enhance learning standards and confidence in patient care ( Chin *et al.*, 2014). It is best represented as a concept of leading edge technology to facilitate human- machine interaction and effectively decrease the gap between realistic and theory based learning by involving the learner in pseudo realistic settings. It differs greatly with respect to its level of development, authenticity, and end user synergy with the virtual background. Comprehending the use of haptic feedback can produce a feeling of resistance when using instruments in a simulated environment. Similar technological principles are being used in training laparoscopic and endoscopic instrumentation to the resident physicians.

#### 4.1 Limitations:

Although SBL can simulate genuine clinical scenarios, it can be a first time practical experience for students requiring coordination, patience, cooperation, and effective guidance. SBL equipment such as mannequins, software and facility areas can be expensive and need appropriate maintenance. Session preparation and arrangement can be time consuming and necessitates enough equipment for equal student opportunity. Professors also need to be suitably trained in operating any gear.

#### 4.2 Recommendations:

The implementation of simulation training alongside the traditional didactic lectures, has shown to reduce errors and improve performance of medical procedures. It is therefore advisable to utilize the simulation technique in teaching complex medical procedures for better patient care outcomes. Simulation-based learning should be implemented at the very start of basic sciences allowing for more hands-on pseudo-clinical exposure. Student enthusiasm can be amplified with smaller group sizes combined with minimal instructional guidance allowing students to independently manage the tasks at hand to increase their participation and peer discussions.

### 5. Social media and video lectures (e-learning)

Social media is a public networking space where end users establish online communities for effective discussion. These online communities are helpful to circulate information, thoughts, and various other contents. There are many social media platforms like Twitter, Facebook, YouTube, and online blogs. Social media has become an elemental part of modern medical societies, hospitals, and advocacy groups. The obligation for an advancing education is more important than ever before, thereby incorporation of social media in the modern educational system is a must. Social media platforms can assist subsidiary traditional knowledge and enhance distant learning. Students and learners of all stratums commonly check the internet for details about diseases, therapies, and associated physiology. Furthermore, many organizations have realized that supporting live-tweeting or blogging of medical conferences as well as dispensing opportunities for wide propagation of content can far outdistance the in-person attendance.

Computer technologies have shown greater impact on medical education, most recently through the electronic distribution of videos. The extensive usage of the vast educational resources available through the internet has significant medical importance. These online resources can be used for practical learning of clinical procedures, demonstrations of anatomical dissections as well as asynchronous learning through online lectures ( Taveira *et al.*, 2016; Sarıhan *et al.*, 2016; Nieder and Nagy, 2002; McNulty *et al.*, 2009). Resources encompass a wide range of subject material ranging from personal homemade videos to specialized content provided by various health care organizations and clinicians for professional education.

#### 5.1 Limitations:

Despite the fact these platforms can supplement and enhance learning, it is important to realize they cannot replace fundamental education and experience. Students are not able to gain the same direct and live contact with teachers with a structured time and location for learning, substantiation of the knowledge as well as interpersonal skills that can only be learned through in-class learning. Also, the information on the platform is not regulated and can easily be misleading.

#### 5.2 Recommendations:

It is advisable to validate and standardize e-learning resources to deliver unbiased, evidence-based, and accurate information on all aspects of healthcare ( Pant *et al.*, 2012). One way to ensure access to suitable online resources is to encourage sharing of the resources between the concerned stakeholders ( Rennie and Morrison, 2013). Reward or compensation can be provided to the academicians who create medical videos and online seminars. Furthermore, the best method of e-learning is to provide a self-paced and blended learning approach which can be achieved through proper collaboration and communication in between the classmates and other experts. This can be achieved through the videoconferencing or other social sites to provide the students with personalized support, target group discussion, and individualized question answer sessions ( Scicluna *et al.*, 2015).

### 6. Peer assisted learning

Peer-assisted learning is the development of knowledge based skill through active help and support of equals. It is a team-based, analogous, non-professional learning framework which comprises a group of motivated people helping each other in the learning process. These participants, tutors and tutees, come from similar educational backgrounds. This learning strategy is conducted through selection of students with suitable characteristics of teaching medical concepts ( Awasthi and Yadav, 2015). This allows for development of capabilities enhancing learning along with practice of medicine. It is an extensive system that promises to ensure strong affiliation amid experiential learning and a collaborative teaching environment.

PAL is helpful to both the tutor and the tutee to enhance their knowledge and understanding. Tutor self-perceived benefits include: skills in expressing thoughts and development in understanding knowledge. Preparing to teach and learn simultaneously and providing feedback to peers have both improved cognitive and non-cognitive benefits to the tutors. It has also shown a greater impact on the assessment scores of the tutees improving their overall academic performance ( Awasthi and Yadav, 2015).

#### 6.1 Limitations:

Tremendous efforts have to be taken in selecting highly efficient tutors to gain the full potential of this learning style. Similarly, perceived stigma associated with peer knowledge, lack of motivation, and willingness for collaboration can be a limiting factor. Students may not be well prepared or have sufficient or accurate knowledge on particular concepts to be able to transmit it to their peers ( Scicluna *et al.*, 2015).

#### 6.2 Recommendations:

Successful implementation of PAL showcases the effectiveness of peer learning; thereby, motivating students for active participation at different levels. It is important to regularly train the students as well as provide them with practice sessions including guidance and assistance ( Ward and Lee, 2005). Peer tutors should develop effective communication skills and sufficient confidence to ensure its success. Assessment, feedback, observations and reflective logging, can be used to monitor both tutor and tutee progress ( Master, Fuchs and Fuchs, 2006). Teachers can assist, modify or provide an alternative learning technique to address the students’ academic needs only if an expected outcome is not achieved.

### 7. Observational learning

Observational learning is learning through demonstration, mostly important in the medical field in due consideration of “patient safety first”. Motor skill development is an essential component of medicinal expertise and therefore must be taught and practiced competently. Numerous medical procedures are termed open-skills as they necessitate physician adaptation to unpredictable and ever-changing environments (e.g. oro-tracheal intubation and surgical suture). The mechanical processing behind this learning strategy comes from mirror neuron systems in the premotor cortex and has a role in the reproduction of observable actions in others ( Cordovani and Cordovani, 2016). The approach to observational learning involves the commitment of the motor system to learn, requiring its implicit engagement by the observer ( Lawrence, Callow and Roberts, 2013). Furthermore, immediate feedback gives the impression that it has an effect not only during physical practice but also during observational learning. However, it shows that aggregates of observational methods and physical practice could be more desirable than physical practice alone.

Observational methods are crucial to learning complex medical procedures enhancing learning and skill through observational practice. Motor skill practice becomes an important attribute to improve performance of medical procedures, and understanding the underlying mechanism for these motor actions play a crucial role in building better training systems. Strategies acquired from this technique lead to flexible capabilities and optimize motivation by enhancing information processing. It also heightens skill development through visual-spatial depiction consequently producing vivid imagery of the working memory.

#### 7.1 Limitations:

Observational learning has some challenges including its implementation into the curriculum. Instructors are not able to control students’ interest in observing a particular technique or skill making it difficult to assess behaviour. Observational sessions are also cumbersome and physically demanding to set up. Similarly, it only provides some of the motor and sensory processes involved in motor skill development compared to actual practice involving the complete process. Students experience the visual input regarding movement execution, without experiencing the neuronal connections to motor periphery or afferent feedback. Moreover, it can introduce observer-bias in which student interest can affect perception and interpretation of the demonstration. Likewise, there can be a transient change in student behaviour introducing a “Hawthorne effect”.

#### 7.2 Recommendations:

Complementation of teaching methodologies can enhance learning and develop clinical acumen. It is therefore important to supplement other forms of teaching methods like observational learning to minimize bias and reduce any gaps seen with a single teaching style ( Van *et al.* 2009). Observational learning does not only have to do with physical demonstrations; dynamic visualizing of demos through video or animations is also beneficial.

### 8. Flipped classroom

Flipped classroom is the newer innovative teaching and learning strategy that incorporates blended learning techniques using online and/or offline instructional content outside the traditional classroom setting. Students are provided with the pre-recorded lectures assigned as homework for class preparation shifting from instructor-centred towards self-directed learning. They solve medical cases by engaging in small groups that will facilitate a team-based approach and promote longer retention of facts ( Hew and Lo, 2018). It also supports student interaction amongst each other to fill the void in their knowledge by acknowledging their diversity and learning strengths.

Flipped classroom promotes self-directed learning, as students are obliged to look into alternative sources to support the given cases. Current research evidence shows that the flipped classroom approach improves student perception, learning, critical thinking skills, and motivation in comparison to traditional lecturing methods (Gilfboy, Heinerichs and Pazzaglia, 2015). Through incorporation of audio-visual tools, students are provided with indefinite access to instructive material thus stimulating an interactive and independent learning experience ( Kellesarian, 2018). Students are able to evaluate their learning, identify their strengths and weaknesses, and are given the possibility to receive feedback and constructive criticism from both their colleagues and teachers to make the necessary improvements in their learning process.

#### 8.1 Limitations:

Despite these advantages, flipped classrooms are criticized for minimal direct instructor involvement and lack of clinical skills collaboration, which are mainly essential for clinical practice. Other challenges include known differences in cognitive abilities that students may use when learning from a screen compared to hard copy material such as comparatively inferior time optimization and distractibility and reduced ability to concentrate ( Khanova *et al.*, 2015). There is a greater need for teachers to thoughtfully prepare and design activities to ensure student preparation and engagement. As evident, neglecting to link pre-learning material to in-class discussions can establish frustration and discontent among students.

#### 8.2 Recommendations:

Successful utilization of flipped classrooms requires an active participation of both the student and teacher. Obtaining feedback by students, encouraging peer learning and promoting pre-reading can further allow smooth operations of flipped classroom. Different strategies like a provision of a quiz at the start of the class can motivate students to pre-read specific topics, address doubts, and ensure reflective learning ( Deshpande *et al.*, 2020).

### 9. Team-based learning

Team-based learning (TBL) is one of the finest learning techniques that recently gained popularity in medical education with the basis of student- centered learning ( Burgess, McGregor and Mellis, 2014). Team-based learning is defined as a learning strategy with a small group of students having the opportunity to apply educational concepts through various activities that comprises critical thinking, individual and team-based tasks, brainstorming followed by immediate feedback from the instructor. TBL has a greater advantage of increasing communication skills and team work strategies in the student groups which are essential for patient care ( O’Daniel and Rosenstein, 2008). Active participation of the student groups was more effectively seen in TBL compared to the PBL. TBL also possesses the major advantage of having students find solutions, make decisions as a team which fosters increased motivation for learning, creates concept mapping and seeds deep learning. Instructors leading small group discussion sessions which mimics team-based learning have shown better student assessment scores compared to the standard didactic lectures ( Arja *et al.*, 2020).

#### 9.1 Limitations:

Unfortunately, there is evidence in which certain students and educators struggle with this learning technique as some students do not value team-work; they find it has reduced effectiveness and efficiency when compared to didactic learning. Certain students value the competitive nature of higher education; this makes some students reluctant to engage in participating and sharing information. Also, problems can lie on knowledge-transfer between students and instructors. Both need to believe in practicing concepts through exercises for proper concept use in real-life situations. Some instructors that do not completely embrace this learning strategy, and revert back to simply telling students information, risk interfering with students’ creative process and critical thinking, causing students to feel less satisfied and feel they haven’t gained sufficient knowledge as they had wanted.

#### 9.2 Recommendations:

To decrease the potential differences in knowledge among the team members when working in groups it is recommended to supplement students with pre-recorded lecture notes and compulsory reading tasks before attending the session with the instructor. Better outcomes can be seen in student groups when the instructor uses open-ended questions to foster discussion among the student groups ( Burgess *et al.*, 2017).

On extensive literature review, few marked multidisciplinary learning techniques are summarized in the below Table 1.

**Table 1.** Review of the literature on multidisciplinary learning techniques summarizing the key research findings

Authors	Research type	Research purpose	Findings
1. Zendejas B1, Brydges R, Wang AT, Cook DA, 2013.	Systematic Review was performed from 10,903 articles screened.	To evaluate patient impact outcomes with the integration of simulation learning.	Simulation-based education was associated with small-to-moderate patient benefits in comparison with no intervention and non-simulation instruction.
2. Xiangyu Ma, Bin Xu, Bin Xu, Qingyun Liu, Yao Zhang, Hongyan Xiong, 2014.	A self-controlled trial was conducted with 251 undergraduate medical students.	To evaluate the effect of integration of EBM into the medical curriculum by measuring undergraduate medical student’s knowledge and personal application.	Integration of EBM into the medical curriculum improved student knowledge, attitudes, and personal application.
3. Mohammad Hadi Imanieh, Seyed Mohsen Dehghani, Ahmad Reza Sobhani, 2014.	An interventional study was conducted among 120 medical students introduced from the Medical Faculty to the Pediatrics Department with no personal involvement.	To evaluate problem-based learning as compared to the contemporary teacher-based medical education or traditional methods.	Problem-based learning method leads to a significant increase in learning and recalling output compared to the traditional method.
4. Annette Burgess, Deborah McGregor, and Craig Mellis, 2014.	Systematic review was done with selection of titles and abstracts based on pre-determined eligibility criteria.	To evaluate the learning benefits in medical tutors and tutees in medical school	PAL showed improved learning benefits in both tutors and tutees.
5. Khadija Qamar, Sabah Rehmanand, Muhammad Alamgir Khan, 2015.	An interventional study was conducted among second-year undergraduate medical students at “Army Medical College” to evaluate case-based learning during small group sessions.	To evaluate the relationship between the effectiveness of small-group sessions with cognitive, motivational, and overall group productivity of the students.	The cognitive and motivational scores were increased after the interactive small group discussion during case-based learning sessions.
6. Cordovani, L. & Cordovani, D. Adv in Health Sci Educ, 2016.	An observational study was conducted among anesthesia teaching and training sessions	To evaluate the observation and physical practice integration in medical techniques.	Combination of observational and physical practice could be better than physical practice alone

## Discussion

Traditionally, lectures occupy the centre of education in pre-clinical years. Didactic lectures have been the formal method of relaying information from instructor to student. However, this approach has been met with numerous challenges necessitating the implementation of modern learning techniques. DL is passive and poorly designed or executed as an active learning exercise for students to be able to effectively enhance their learning through collaboration ( White *et al.*, 2014). Students are becoming increasingly reluctant to engage in teaching and learning beyond the realm of the classroom. Modern approaches to learning are student-centred and focus the responsibility of learning on the learners ( Ramnanan and Pound, 2017). Unparallel progression in the medical systems dictates the need for an educational system that actively engages future students. Students more than just listen; they are continually engaged in the learning process through active participation ( Luscombe and Montgomery, 2016). They are more effective in improving knowledge and understanding in medical education compared to didactic lectures, and shown to improve long-term retention of knowledge and self-directed learning skills ( Hernández-Torrano, Ali and Chan, 2017).

Modern techniques of learning medicine are imperative in medical education. Integrating modern techniques of learning in medical education addresses the differences in learning style preferences that can affect students’ performance in various aspects of basic medical sciences ( Inra *et al.,* 2017). These techniques, such as CBL, EBM and PBL, motivate studying by actively involving students and linking theory to real life situations. They improve competency, logical thinking and better clinical reasoning. For example, PAL helps students express their thoughts and share their knowledge to develop understanding. Observational learning allows for immediate feedback and enhances performance of medical procedures which are almost always a daily routine of physicians. Modern techniques of learning also provide freedom to explore knowledge and give an opportunity for reflection in a controlled environment. In simulation studies, student mistakes can be acceptable as they are not deleterious to the patient and the mistakes serve as reinforcement in avoiding future medical errors. Similarly, the internet is an effective tool in providing facts about disease processes, therapies, and management. Through social networking, online collaboration is promoted and content-specific information is easily disseminated to students in particular subgroups. YouTube videos from reliable sources have proven to provide medical students with a wealth of valuable information particularly on how to perform various clinical examinations.

The process of learning is a natural, active, intentionally mediated and highly personal process in which there is constructing meaning from information and life experiences ( McCombs, 1991). This concept suggests that individuals have a natural propensity for learning surrounded by the correct motivational environment as well as that the learning process is highly personal. A motivated student is therefore a lifelong learner and vice versa. Both learning and motivation for seeking knowledge are natural qualities and are influenced by a person’s view of themselves, their goals and expectations. Lifelong learning is also a natural human capacity strengthened by the discovery of one’s learning tendencies. Accordingly, it is important to promote self-initiating learning techniques as they are the most persistent and permeative ( Collins, 2004). It should be relevant to the students’ work and obligations valued by them. Facilitators should be explicit in the learning objectives and how that particular activity would allow them to achieve their goals. Educators should also be aware of each student’s needs and devise activities that meet those needs with emphasis on motivation. Creating a motivational atmosphere can involve establishment of social relationships (i.e. making friends), fulfilment of expectations, professional growth, and cognitive benefit. The optimal learning experience is a self-motivated and active process, with encouragement of student participation, sharing of experiences, and involvement in discussions.

There is a greater need for curricular integration to complement basic and clinical sciences to enhance learning and promote student engagement. Early introduction and exposure of various teaching-learning strategies enhances understanding and aids in clinical practice. These learning methods attend to individual learning differences and integrate various learning strategies allowing students. Currently, medical colleges have realized the value of early clinical exposure to support vertical integration in preclinical studies. Beyond doubt, the incorporation of modern learning methods will facilitate the acquisition of knowledge and skills. This is in contrast tothe traditional medical curriculum which focuses entirely on the core medical facts rather than an overall development in research and innovation. A regular review and implementation of the modern teaching-learning technologies is needed along with timely detection of a dysfunctional curriculum ( Chang, 2016). Concerned stakeholders should come together and play an active role in formulation and design of the curriculum intended to ensure an improvement in medical students’ aptitude, attitude and future application of medical knowledge. As such, the educational institutions should design, introduce and implement these alternative teaching-learning strategies early in the curriculum for an optimal teaching and learning environment. This provides students the freedom to explore and reflect on their knowledge as well as help in expanding and modernizing medical education.

## Conclusion

Learning is a contiguous process and it is important to recognize that students have different styles of learning. Some of these modern teaching learning methods in medical education include CBL, EBM, PBL, SBL, e-learning, PAL, observational learning, flipped classroom model and team-based learning. These student-centred alternative teaching and learning techniques broaden students’ thinking through creative new approaches in constructive knowledge acquisition and strengthens the professional expertise by developing skills, competency, and leadership in the medical field. Medical education, therefore, should be flexible enough to accommodate and incorporate multidisciplinary teaching models effectively and appropriately at the right moment and context beginning from the preclinical years.

## Abbreviations

DL: Didactic lectures

CBL: Case based learning

PBL: Problem based learning.

EBM: Evidence based medicine

SBL: Simulation based learning

PAL: Peer assisted learning

TBL: Team based learning

## Take Home Messages

• Didactic learning with minimal student engagement has warranted student-centered multidisciplinary teaching methods at present.
• Modern techniques of learning are necessary to promote active student participation.
• Modern techniques of learning like evidence-based medicine, simulation and problem-based learning are a critical tool for developing analytical and problem solving skills.
• Early introduction and exposure of alternative learning techniques facilitate vertical integration, and acquisition of knowledge and skills for the preparation of clinical practice.
• Medical education should be flexible to accommodate and incorporate diverse teaching and learning techniques into the medical curriculum.

## Notes On Contributors

**Krishna Teja Challa**, MD Student, is a medical student at St.Lukes Pheonix Hospital and Mountain Vista Medical Centre with experience in Pediatrics, Internal Medicine, and Family Medicine departments. He is also a researcher in the Medical Department at Avalon University School of Medicine. His research interests include public health, infectious disease, preventative care, and medical education. ORCID ID: https://orcid.org/0000-0001-5881-6852

**Abida Sayed**, MD Student, BSc(Hons), is a medical student at Beckley Appalachian Regional Healthcare and Raleigh General Hospital. She currently has experience in the Psychiatry and Pediatrics Departments and is a researcher and committee member of the Research Department at Avalon University School of Medicine. Her research interests are carcinogenesis and oncology, public health and prevention as well as medical education. ORCID ID: https://orcid.org/0000-0002-7323-0793

**Yogesh Acharya**, MD, FAcadMEd, is a research fellow at Western Vascular Institute, Department of Vascular and Endovascular Surgery, University Hospital, National University of Ireland, Galway, Ireland. He has great experience of teaching medical students in Pathology, Epidemiology, and Microbiology for more than 10 years. ORCID ID: https://orcid.org/0000-0003-1829-5911
