# Supplementary material for: Modern techniques of teaching and learning in medical education: a descriptive literature review
Source: MedEdPublish (2016). 2021 Jan 21;10:18. [Version 1] doi: 10.15694/mep.2021.000018.1 (PMC10939590; doi:10.15694/mep.2021.000018.1)
Supplement: Peer review history

# Reviewer report: Reviewer response for version 1

**DOI:** 10.21956/mep.18837.r26838

**Published:** 2021-01-28

**Authors:** Simonetta Ausoni

**Affiliations:** 1 Department of Biomedical Sciences- University of Padova- School of Medicine

This review has been migrated. The reviewer awarded 4 stars out of 5 This is a useful and interesting review that provides the reader with an overview of the most effective and most innovative teaching techniques in medical education. For each of them the authors discuss limitations, advantages and possible suggestions in order to choose the appropriate ones to design learning activities tailored to the courses. I too appreciated the fact that it was written by medical students, an excellent example of active collaboration in the process of teaching improvement.

Reviewer Expertise:

NA

No decision status is available

# Reviewer report: Reviewer response for version 1

**DOI:** 10.21956/mep.18837.r26837

**Published:** 2021-01-25

**Authors:** BALAJI ARUMUGAM

**Affiliations:** 1 TAGORE MEDICAL COLLEGE AND HOSPITAL

This review has been migrated. The reviewer awarded 4 stars out of 5 Thanks a lot to the authors for publishing this wonderful comprehensive article on modern teaching techniques which highlights the importance and challenges of modern learning systems. Case-based learning, evidence-based medicine, problem-based learning, simulation-based learning, e-learning, peer-assisted learning, observational learning, flipped classroom and team based learning are some of the modern learning methodologies. The most fascinating method to me as a medical educator is having all the newer methods in the same article explaining the method of utilizing, limitations etc was presented nicely in this article.

Reviewer Expertise:

NA

No decision status is available

# Reviewer report: Reviewer response for version 1

**DOI:** 10.21956/mep.18837.r26839

**Published:** 2021-01-24

**Authors:** Balakrishnan Nair

**Affiliations:** 1 Centre for Medical Professional Development and University of Newcastle

This review has been migrated. The reviewer awarded 4 stars out of 5 This is a good review of the current learning methods. It is good to see that the review is done my medical students. This underlies that it is all about learning and not teaching. All teaching should be learner centred. The conclusion is learners are a heterogenous group and different learning styles suit different type of learners. This should be the core principle of all curricula

Reviewer Expertise:

NA

No decision status is available
